# Normal forms of dispersive scalar Poisson brackets with two independent variables

**DOI:** 10.1007/s11005-018-1076-x

**Published:** 2018-03-26

**Authors:** Guido Carlet, Matteo Casati, Sergey Shadrin

**Affiliations:** 1 IMB UMR 5584 CNRS Université Bourgogne Franche-Compté, 21000 Dijon, France; 2 Istituto Nazionale d’Alta Matematica, Rome, Italy; 3 Department of Mathematical Sciences, Loughborough University, Loughborough, LE11 3TU UK; 4 Korteweg-de Vries Instituut voor Wiskunde, Universiteit van Amsterdam, Postbus 94248, 1090 GE Amsterdam, The Netherlands

**Keywords:** Poisson brackets, Poisson cohomology, Hamiltonian operator, Miura transformation, 37K05

## Abstract

We classify the dispersive Poisson brackets with one dependent variable and two independent variables, with leading order of hydrodynamic type, up to Miura transformations. We show that, in contrast to the case of a single independent variable for which a well-known triviality result exists, the Miura equivalence classes are parametrised by an infinite number of constants, which we call numerical invariants of the brackets. We obtain explicit formulas for the first few numerical invariants.

## Introduction

Let $\mathcal {A}$ be the space of differential polynomials in the variable *u*, i.e. formal power series in the variables $\partial _{x^1}^{k_1}\partial _{x^2}^{k_2} u$ with coefficients which are smooth functions of *u*:

$$\begin{aligned} \mathcal {A}= C^\infty (U) \left[ \left[ \left\{ u^{(k_1,k_2)} =\partial _{x^1}^{k_1} \partial _{x^2}^{k_2} u \text { with } k_1, k_2 \geqslant 0 , \,(k_1,k_2 ) \not = (0,0) \right\} \right] \right] , \end{aligned}$$

for $U \subset \mathbb {R}$. The standard degree $\deg$ on $\mathcal {A}$ counts the number of derivatives $\partial _{x^1}$, $\partial _{x^2}$ in a monomial, i.e. it is defined by $\deg ( \partial _{x^1}^{k_1} \partial _{x^2}^{k_2} u) = k_1 + k_2$.

In this paper, we classify, up to Miura transformations, the dispersive Poisson brackets with one dependent variable *u* and two independent variables $x^1$, $x^2$ of the form

1 $$\begin{aligned}&\left\{ u\left( x^1,x^2\right) ,u\left( y^1,y^2\right) \right\} \nonumber \\&\quad = \left\{ u\left( x^1,x^2\right) , u\left( y^1,y^2\right) \right\} ^0 \nonumber \\&\qquad \quad +\,\sum _{k>0} \epsilon ^k \sum _{\begin{array}{c} k_1,k_2 \geqslant 0 \\ k_1 +k_2 \leqslant k+1 \end{array}} A_{k; k_1, k_2}(u(x)) \delta ^{(k_1)}\left( x^1-y^1\right) \delta ^{(k_2)}\left( x^2-y^2\right) \qquad \end{aligned}$$

where $$A_{k; k_1,k_2} \in \mathcal {A}$$ and $$\deg A_{k; k_1,k_2} = k - k_1 - k_2 +1$$.

The leading term $\{ u(x^1,x^2) , u(y^1,y^2) \}^0$ is a (scalar, two-dimensional) Poisson bracket of Dubrovin–Novikov (or hydrodynamic) type [12, 13], and in other words it is of the form

$$\begin{aligned}&\left\{ u\left( x^1,x^2\right) , u\left( y^1,y^2\right) \right\} ^0\\&\quad = \sum _{i=1}^2 \left[ g^i(u(x)) \partial _{x^i} + b^i(u(x)) \partial _{x^i} u(x) \right] \delta \left( x^1-y^1\right) \delta \left( x^2-y^2\right) , \end{aligned}$$

which we assume to be non-degenerate.

The conditions imposed on the functions $g^i(u)$ and $b^i(u)$ by the requirement that $\{ , \}^0$ is skew-symmetric and satisfies the Jacobi identity have been studied by several authors [15, 21, 22]. We require the additional condition that the bracket is non-degenerate, namely that the bracket does not vanish for any value of the function *u*(*x*). In the specific case considered here, where there is a single dependent variable and two independent variables, such conditions guarantee the existence of a change of coordinates in the dependent variable (a Miura transformation of the first kind), to a flat coordinate that we still denote with *u*, in which the bracket assumes the form

$$\begin{aligned} \left\{ u\left( x^1,x^2\right) , u\left( y^1,y^2\right) \right\} ^0&= c^1 \delta ^{(1)}\left( x^1-y^1\right) \delta \left( x^2-y^2\right) \\&\quad \,\, +\, c^2 \delta \left( x^1-y^1\right) \delta ^{(1)}\left( x^2-y^2\right) . \end{aligned}$$

We can moreover perform (see [2]) a linear change in the independent variables $x^1$, $x^2$ such that the Poisson bracket assumes the standard form

2 $$\begin{aligned} \left\{ u\left( x^1,x^2\right) , u\left( y^1,y^2\right) \right\} ^0 = \delta \left( x^1-y^1\right) \delta ^{(1)}\left( x^2-y^2\right) . \end{aligned}$$

The Miura transformations (of the second kind [18]) are changes of variable of the form

3 $$\begin{aligned} v = u + \sum _{k\geqslant 1} \epsilon ^k F_k \end{aligned}$$

where $F_k \in \mathcal {A}$ and $\deg F_k= k$. They form a group called Miura group. We say that two Poisson brackets which are mapped to each other by a Miura transformation are Miura equivalent.

As follows from the discussion so far, the classification of dispersive Poisson brackets of the form (1) (with non-degeneracy condition) under Miura transformations (3), diffeomorphisms of the dependent variable and linear changes of the independent variables reduces to the problem of finding the normal forms of the equivalence classes under Miura transformations of the second kind (3) of the Poisson brackets (1) with leading term (2).

We solve this problem in our main result:

### Theorem 1

The normal form of Poisson brackets (1) with leading term (2) under Miura transformations of the second kind is given by

4 $$\begin{aligned} \left\{ u\left( x^1,x^2\right) ,u\left( y^1,y^2\right) \right\}&= \delta \left( x^1-y^1\right) \delta ^{(1)}\left( x^2-y^2\right) \nonumber \\&\quad \, + \sum _{k \geqslant 1} \epsilon ^{2k+1} c_k \delta ^{(2k+1)}\left( x^1-y^1\right) \delta \left( x^2-y^2\right) \end{aligned}$$

for a sequence of constants $c=(c_1, c_2, \ldots )$.

### Remark 1

By “normal form”, in the main theorem, we mean that:

i. for any choice of constants $c_k$ formula (4) defines a Poisson bracket which is a deformation of (2);
ii. two Poisson brackets of the form (4) are Miura equivalent if and only if they are defined by the same constants $c_k$;
iii. and any Poisson bracket of the form (1) can be brought to the normal form (4) by a Miura transformation.

We call the constants $c_k$ the *numerical invariants* of the Poisson bracket.

### Example 1

(Hamiltonian structure of KP equation, [8]) Kadomtsev–Petviashvili (KP) equation describes two-dimensional shallow water waves, being a generalisation of $K\mathrm{d}V$ equation. In its standard form, it is a $(2+1)$-dimensional PDE for a scalar field

$$\begin{aligned} \partial _x\left( u_t+6uu_x+u_{xxx}\right) =u_{yy} \end{aligned}$$

KP equation is Hamiltonian and integrable [24]; it is generally treated as the compatibility condition of an integrable $(1+1)$-dimensional hierarchy, where both the *t* and *y* coordinates play the role of times. However, it is possible to cast the equation in evolutionary form, with the introduction of the inverse derivative operator $\partial _x^{-1}$. KP equation can be written as

$$\begin{aligned} u_t=\partial _x^{-1}u_{yy}-\partial _{x}\left( 3u^2+u_{xx}\right) \end{aligned}$$

which is Hamiltonian with respect to the Hamiltonian functional

$$\begin{aligned} H=\int \left( \frac{\left( \partial _x^{-1}u_y\right) ^2}{2}+\frac{u_x^2}{2}-u^3\right) \ \end{aligned}$$

and the Poisson bracket

5 $$\begin{aligned} \{u(x,y),u(w,z)\}=\delta ^{(1)}(x-w)\delta (y-z). \end{aligned}$$

The Poisson bracket (5) is of the form (4) for $c_k\equiv 0$ and the relabeling of the independent variables $(x^1\rightarrow y,x^2\rightarrow x,y^1\rightarrow z, y^2\rightarrow w)$.

The deformation theory of Hamiltonian—and, albeit not addressed in our paper, bi-Hamiltonian—structures plays an important role in the classification of integrable Hamiltonian PDEs [10, 14]. Most results in this field have been obtained for $(1+1)$-dimensional systems, namely the ones that depend only on one space variable.

The main result in this line of research is the triviality theorem [9, 14, 16] of Poisson brackets of Dubrovin–Novikov type. Together with the classical results by Dubrovin and Novikov [12], this allows to conclude that the dispersive deformations of non-degenerate Dubrovin–Novikov brackets are classified by the signature of a pseudo-Riemannian metric. Similarly, deformations of bi-Hamiltonian pencils [1, 20] are parametrised by functions of one variable, the so-called central invariants [10, 11]; in a few special cases, the corresponding bi-Hamiltonian cohomology has been computed, in particular for scalar brackets [4, 5, 19], and in the semi-simple *n*-component case [3, 6] . The $(2+1)$-dimensional case is much less studied: the classification of the structures of hydrodynamic type has been completed up to the four-component case [15], and the nontriviality of the Poisson cohomology in the two-component case has been established [7]. In our recent paper [2] we computed the Poisson cohomology for scalar—namely, one-component—brackets. Since such a cohomology is far from being trivial, the actual classification of the dispersive deformations of such brackets is a highly complicated task. We address and solve it in the present paper.

The outline of the paper is as follows: in Sect. 1 we quickly recall basic definitions and facts related with the theta formalism. In Sect. 2, we specialise some results from our previous work [2] to the $D=2$ case to obtain an explicit description of the second Poisson cohomology. In Sect. 3, we prove our main result. The proof is split into three steps corresponding to the three parts in Remark 1. In Sect. 4.4, we prove some technical lemmas that are required in the proof of Proposition 2. Finally, in Sect. 4 we give an explicit expression of the first few numerical invariants of the Poisson bracket.

## Theta formalism

We present here a short summary of the basic definitions of the theta formalism for local variational multivector fields, specialising the formulas to the scalar case with two independent variables, i.e. $N=1$, $D=2$. We refer the reader to [2] for the general *N*, *D* case.

Let $\mathcal {A}$ be the space of differential polynomials

$$\begin{aligned} \mathcal {A}= C^\infty (\mathbb {R}) \left[ \left[ \left\{ u^{(s,t)} , s, t \geqslant 0 , \ (s, t) \not = (0,0) \right\} \right] \right] , \end{aligned}$$

where we denote $u^{(s,t)} = \partial _x^s \partial _y^t u$, and $C^\infty (\mathbb {R})$ denotes the space of smooth functions in the variable *u*. The standard gradation $\deg$ on $\mathcal {A}$ is given by $\deg u^{(s,t)} = s+t$. We denote $\mathcal {A}_d$ the homogeneous component of degree *d*.

Using the standard derivations $\partial _x$ and $\partial _y$ on $\mathcal {A}$, we define the space of local functionals as

$$\begin{aligned} \mathcal {F}= \frac{\mathcal {A}}{\partial _{x} \mathcal {A}+ \partial _{y}\mathcal {A}} , \end{aligned}$$

and the projection map from $\mathcal {A}$ to $\mathcal {F}$ is denoted by a double integral, which associates to $f\in \mathcal {A}$ the element

$$\begin{aligned} \int f \ \mathrm{d}x \ \mathrm{d}y \end{aligned}$$

in $\mathcal {F}$. Moreover, we will denote by the partial integrals $\int \mathrm{d}x$ , $\int \mathrm{d}y$ the projections from $\mathcal {A}$ to the quotient spaces $\mathcal {A}/\partial _{x}\mathcal {A}$, $\mathcal {A}/\partial _{y}\mathcal {A}$.

The variational derivative of a local functional $F=\int f$ is defined as

$$\begin{aligned} \frac{\delta F}{\delta u} = \sum _{s,t \geqslant 0} (- \partial _x)^s (- \partial _y)^t\frac{\partial f}{\partial u^{(s,t)}} . \end{aligned}$$

A local *p*-vector *P* is a linear *p*-alternating map from $\mathcal {F}$ to itself of the form

$$\begin{aligned} P(I_1, \ldots ,I_p) = \int P_{(s_1,t_1), \ldots , (s_p,t_p)} \ \partial _x^{s_1} \partial _y^{t_1} \left( \frac{\delta I_1}{\delta u} \right) \cdots \partial _x^{s_p} \partial _y^{t_p} \left( \frac{\delta I_p}{\delta u} \right) \ \mathrm{d}x \ \mathrm{d}y \end{aligned}$$

where $P_{(s_1,t_1), \ldots , (s_p,t_p)} \in \mathcal {A}$, for arbitrary $I_1, \ldots , I_p \in \mathcal {F}$. We denote the space of local *p*-vectors by $\Lambda ^p \subset \mathrm {Alt}^p(\mathcal {F}, \mathcal {F})$.

Clearly an expression of the form (1) defines a local bivector by the usual formula

$$\begin{aligned} \{ I_1, I_2 \} = \int \frac{\delta I_1}{\delta u(x^1, y^1)} \left\{ u(x^1, y^1), u(x^2,y^2) \right\} \frac{\delta I_2}{\delta u(x^2,y^2)} \ \mathrm{d}x^1 \ \mathrm{d}y^1 \ \mathrm{d}x^2 \ \mathrm{d}y^2 \end{aligned}$$

which equals to

$$\begin{aligned} \int \sum _{k\geqslant 0} \epsilon ^k \frac{\delta I_1}{\delta u(x,y)} \sum _{\begin{array}{c} s,t \geqslant 0 \\ s +t \leqslant k+1 \end{array}} A_{k; s, t}(u(x)) \partial _x^s \partial _y^t \frac{\delta I_2}{\delta u(x,y)} \ \mathrm{d}x \ \mathrm{d}y. \end{aligned}$$

The theta formalism, introduced first in the context of formal calculus of variations in [16], can be easily extended to the multidimensional setting [2] and allows to treat the local multivectors in a more algebraic fashion.

We introduce the algebra $\hat{\mathcal {A}}$ of formal power series in the commutative variables $u^{(s,t)}$ and anticommuting variables $\theta ^{(s,t)}$, with coefficients given by smooth functions of *u*, i.e.

$$\begin{aligned} \hat{\mathcal {A}}:= C^\infty (\mathbb {R}) \left[ \left[ \left\{ u^{(s,t)},(s,t)\not =(0,0) \right\} \cup \left\{ \theta ^{(s,t)} \right\} \right] \right] . \end{aligned}$$

The standard gradation $\deg$ and the super-gradation $\deg _\theta$ of $\hat{\mathcal {A}}$ are defined by setting

$$\begin{aligned} \deg u^{(s,t)} =\deg \theta ^{(s,t)} = s+t, \quad \deg _\theta u^{(s,t)} = 0 , \quad \deg _\theta \theta ^{(s,t)} = 1 . \end{aligned}$$

We denote $\hat{\mathcal {A}}_d$, resp. $\hat{\mathcal {A}}^p$, the homogeneous components of standard degree *d*, resp. super-degree *p*, while $\hat{\mathcal {A}}_d^p := \hat{\mathcal {A}}_d \cap \hat{\mathcal {A}}^p$. Clearly $\hat{\mathcal {A}}^0 =\mathcal {A}$. The derivations $\partial _x$ and $\partial _y$ are extended to $\hat{\mathcal {A}}$ in the obvious way.

We denote by $\hat{\mathcal {F}}$ the quotient of $\hat{\mathcal {A}}$ by the subspace $\partial _{x} \hat{\mathcal {A}}+ \partial _{y} \hat{\mathcal {A}}$, and by a double integral $\int \mathrm{d}x \ \mathrm{d}y$ the projection map from $\hat{\mathcal {A}}$ to $\hat{\mathcal {F}}$. Since the derivations $\partial _x$, $\partial _y$ are homogeneous, $\hat{\mathcal {F}}$ inherits both gradations of $\hat{\mathcal {A}}$.

It turns out, see Proposition 2 in [2], that the space of local multivectors $\Lambda ^p$ is isomorphic to $\hat{\mathcal {F}}^p$ for $p\not =1$, while $\Lambda ^1$ is isomorphic to the quotient of $\hat{\mathcal {F}}^1$ by the subspace of elements of the form $\int (k_1 u^{(1,0)} + k_2 u^{(0,1)} ) \theta$ for two constants $k_1$, $k_2$. Moreover $\hat{\mathcal {F}}^1$ is isomorphic to the space ${{\mathrm{Der}}}'(\mathcal {A})$ of derivations of $\mathcal {A}$ that commute with $\partial _x$ and $\partial _y$.

The Schouten–Nijenhuis bracket

$$\begin{aligned}{}[,]:\hat{\mathcal {F}}^p \times \hat{\mathcal {F}}^q \rightarrow \hat{\mathcal {F}}^{p+q-1} \end{aligned}$$

is defined as

$$\begin{aligned}{}[P , Q ] = \int ^D \left( \frac{\delta P}{\delta \theta } \frac{\delta Q}{\delta u} + (-1)^p \frac{\delta P}{\delta u} \frac{\delta Q}{\delta \theta } \right) \ \mathrm{d}x \ \mathrm{d}y , \end{aligned}$$

where the variational derivative with respect to $\theta$ is defined as

$$\begin{aligned} \frac{\delta }{\delta \theta } = \sum _{s,t\geqslant 0} (-\partial _x)^s (-\partial _y)^t \frac{\delta }{\delta \theta ^{(s,t)}} . \end{aligned}$$

It is a bilinear map that satisfies the graded symmetry

$$\begin{aligned}{}[P,Q] = (-1)^{pq} [Q,P] \end{aligned}$$

and the graded Jacobi identity

$$\begin{aligned} (-1)^{pr} [[P,Q],R] + (-1)^{qp} [[Q,R],P] + (-1)^{rq} [[R,P],Q] =0 \end{aligned}$$

for arbitrary $P\in \hat{\mathcal {F}}^p$, $Q\in \hat{\mathcal {F}}^q$ and $r\in \hat{\mathcal {F}}^r$.

A bivector $P\in \hat{\mathcal {F}}^2$ is a Poisson structure when $[P,P]=0$. In such case $d_P := \mathrm{ad}_P = [P, \cdot ]$ squares to zero, as a consequence of the graded Jacobi identity, and the cohomology of the complex $(\hat{\mathcal {F}}, d_P)$ is called Poisson cohomology of *P*.

The Miura transformations of the second kind [18] are changes of variable of the form

$$\begin{aligned} u \mapsto \tilde{u} =\sum _{k=0}^\infty \epsilon ^k F_k(u) \end{aligned}$$

on the space $\mathcal {A}$, where $F_k\in \mathcal {A}_k$. They form a subgroup of the general Miura group [14] which also contains the diffeomorphisms of the variable *u*. The action of a general Miura transformation of the second kind on a local multivector *Q* in $\hat{\mathcal {F}}$ is given by the exponential of the adjoint action with respect to the Schouten–Nijenhuis bracket

$$\begin{aligned} e^{\mathrm {ad}_X}Q=Q+[X,Q]+\frac{1}{2}[X,[X,Q]]+\frac{1}{6}[X,[X,[X,Q]]]+\cdots , \end{aligned}$$

where $X\in \hat{\mathcal {F}}^1_{\geqslant 1}$ is a local vector field such that $e^{\mathrm {ad}_X} u = \tilde{u}$.

## Poisson cohomology

In our previous paper [2], we gave a description of the Poisson cohomology of a scalar multidimensional Poisson bracket in terms of the cohomology of an auxiliary complex with constant coefficients. Our aim here is to give an explicit description of a set of generators of the Poisson cohomology in the $D=2$ case, which will be used in the proof of the main theorem in the next Section.

Let us begin by recalling without proof a few results from our paper [2], specialising them to the case $D=2$.

Consider the short exact sequences of differential complexes

6 $$\begin{aligned}&0 \rightarrow \hat{\mathcal {A}}/ \mathbb {R}\xrightarrow {\partial _x} \hat{\mathcal {A}}\xrightarrow {\int \mathrm{d}x} \hat{\mathcal {F}}_1 \rightarrow 0, \end{aligned}$$

7 $$\begin{aligned}&0 \rightarrow \hat{\mathcal {F}}_1 / \mathbb {R}\xrightarrow {\partial _y} \hat{\mathcal {F}}_1 \xrightarrow {\int \mathrm{d}y} \hat{\mathcal {F}}\rightarrow 0 , \end{aligned}$$

where the differential is induced in all spaces by

$$\begin{aligned} \varDelta = \sum _{s,t \geqslant 0} \theta ^{(s,t+1)} \frac{\partial }{\partial u^{(s,t)}}. \end{aligned}$$

On $\hat{\mathcal {F}}$ such differential coincides with $\mathrm {ad}_{\mathfrak {p}_1}$, where $\mathfrak {p}_1=\frac{1}{2}\int \theta \theta ^{(0,1)}\mathrm{d}x\mathrm{d}y$.

In the long exact sequence in cohomology associated with (6), the Bockstein homomorphism vanishes; therefore,

$$\begin{aligned} H(\hat{\mathcal {F}}_1) = \frac{H(\hat{\mathcal {A}})}{\partial _x H(\hat{\mathcal {A}})}. \end{aligned}$$

Moreover, the cohomology classes in $H(\hat{\mathcal {A}})$ can be uniquely represented by elements of the polynomial ring $\varTheta$ generated by the anticommuting variables $\theta ^{(s,0)}$, $s\geqslant 0$ with real coefficients.

The map induced in cohomology by the map $\partial _y$ in the short exact sequence (7) vanishes; therefore, we get the following exact sequence

8 $$\begin{aligned} 0 \rightarrow \left( \frac{\varTheta }{\partial _x \varTheta }\right) ^p_d \xrightarrow {\int \mathrm{d}y} H^p_d(\hat{\mathcal {F}}) \rightarrow \left( \frac{\varTheta }{\partial _x \varTheta }\right) ^{p+1}_d \rightarrow 0 , \end{aligned}$$

where the third arrow is the Bockstein homomorphism.

This sequence allows us to write the Poisson cohomology $H^p(\hat{\mathcal {F}})$ as a sum of two homogeneous subspaces of $\varTheta \slash \partial _x \varTheta$ in super-degree *p* and $p+1$, respectively, where the first one is simply injected, while the second one has to be reconstructed via the inverse to the Bockstein homomorphism.

Let $\iint a \ \mathrm{d}x \ \mathrm{d}y \in \hat{\mathcal {F}}^p_d$ be an $\mathrm {ad}_{\mathfrak {p}_1}$-cocycle. Then, there exist $b, b' \in \hat{\mathcal {A}}^{p+1}_d$ such that

$$\begin{aligned} \varDelta a = \partial _y b + \partial _x b'. \end{aligned}$$

The Bockstein homomorphism assigns to the cocycle $\iint a \ \mathrm{d}x \ \mathrm{d}y$ the cocycle $\int b \ \mathrm{d}x \in \hat{\mathcal {F}}^{p+1}_d$.

Let us define a map $\mathcal {B}: \varTheta \rightarrow \hat{\mathcal {A}}$ by

9 $$\begin{aligned} \mathcal {B}= \sum _{i\geqslant 0}u^{(i,0)}\frac{\partial }{\partial \theta ^{(i,0)}}, \end{aligned}$$

which clearly commutes with $\partial _x$, and therefore induces a map from $\frac{\varTheta }{\partial _x \varTheta }$ to $\hat{\mathcal {F}}$. We have that

$$\begin{aligned} \varDelta \mathcal {B}= \partial _y , \end{aligned}$$

and consequently, $\mathcal {B}$ defines a splitting map

$$\begin{aligned} \mathcal {B}: \left( \frac{\varTheta }{\partial _x \varTheta }\right) ^{p+1}_d \rightarrow H^p_d(\hat{\mathcal {F}}) \end{aligned}$$

for the short exact sequence (8).

We have therefore shown that

### Lemma 1

$${H}^{p}_{d}(\hat{\mathcal {F}}) = \left( \frac{\varTheta }{\partial _x \varTheta }\right) ^{p}_d \oplus \mathcal {B}\left( \frac{\varTheta }{\partial _x \varTheta }\right) ^{p+1}_d .$$

We remark that this lemma gives an explicit description of representatives of the cohomology classes in $H^p_d(\hat{\mathcal {F}})$. In particular, the only non-trivial classes in $\varTheta \slash \partial _x \varTheta$ in super-degree $p=2$ are given by $\theta \theta ^{(2k+1,0)}$ for $k\geqslant 1$ and correspond to the deformations of the Poisson brackets in Theorem 1. The following reformulation of this observation will be useful in the proof of Proposition 2:

### Corollary 1

$$\begin{aligned}&\displaystyle H^2_{2k}(\hat{\mathcal {F}}) = \mathcal {B}\left( \frac{\varTheta }{\partial _x \varTheta }\right) ^{3}_{2k}, \\&\displaystyle H^2_{2k+1}(\hat{\mathcal {F}}) = \mathbb {R}\theta \theta ^{(2k+1,0)} \oplus \mathcal {B}\left( \frac{\varTheta }{\partial _x \varTheta }\right) ^{3}_{2k+1}. \end{aligned}$$

Moreover, we can define an explicit basis of $\left( \frac{\varTheta }{\partial _x \varTheta }\right) ^{3}_{d}$ and $\mathcal {B}\left( \frac{\varTheta }{\partial _x \varTheta }\right) ^{3}_{d}$:

### Lemma 2

A basis of $\left( \frac{\varTheta }{\partial _x \varTheta }\right) ^{3}_{d}$ is given by representatives

$$\begin{aligned}&\theta ^{k-l} \theta ^{k-l-1} \theta ^{2l} , \quad l=0,\ldots , \left\lfloor \frac{k-2}{3} \right\rfloor , \quad \text {for } d=2k-1 , \\&\theta ^{k-l} \theta ^{k-l-1} \theta ^{2l+1} , \quad l=0,\ldots , \lfloor \frac{k-3}{3} \rfloor , \quad \text {for } d=2k, \end{aligned}$$

where we use the notation $\theta ^k = \theta ^{(k,0)}$.

### Proof

More generally we can prove that a basis of $\left( \frac{\varTheta }{\partial _x \varTheta }\right) ^{p}_{d}$ is given by

10 $$\begin{aligned} \theta ^{i_2+1} \theta ^{i_2} \theta ^{i_3} \cdots \theta ^{i_p} \end{aligned}$$

with

$$\begin{aligned} i_2> i_3>\cdots > i_p \geqslant 0, \quad 1+ 2i_2 + i_3 + \cdots + i_p = d. \end{aligned}$$

A basis of $\varTheta ^p_d$ is given by monomials $\theta ^{i_1} \cdots \theta ^{i_p}$ with

$$\begin{aligned} i_1> i_2>\cdots > i_p \geqslant 0 , \quad i_1 + \cdots +i_p = d. \end{aligned}$$

We arrange such monomials in *lexicographic order*, that is, we say that $\theta ^{i_1} \cdots \theta ^{i_p} > \theta ^{j_1} \cdots \theta ^{j_p}$ if $i_1 > j_1$, or if $i_1 = j_1$ and $i_2 > j_2$, and so on.

For an element $a = \theta ^{i_1} \cdots \theta ^{i_p}$ of the basis of $\varTheta ^p_{d-1}$, we have that the leading term (in lexicographic order) of $\partial _x a$ is given by

11 $$\begin{aligned} (\partial _x a)^{\mathrm{top}}=\theta ^{i_1+1} \theta ^{i_2} \cdots \theta ^{i_p} . \end{aligned}$$

Note that if $a_1 > a_2$, then $(\partial _x a_1)^{\mathrm{top}} > (\partial _x a_2)^{\mathrm{top}}$. This implies that the images $\partial _x a$ of the monomials $a \in \varTheta ^p_{d-1}$ are linearly independent in $\varTheta _d^p$. Given a representative of a class in $\left( \frac{\varTheta }{\partial _x \varTheta }\right) ^{p}_{d}$, we can express all the monomials of the form (11) in terms of combinations of monomials of strictly lower lexicographic order. It follows that a basis can be chosen in the form (10).

By specialising to the case $p=3$, and spelling out the allowed sets of indexes, we obtain the statement of the lemma. $\square$

It follows that a basis of $\mathcal {B}\left( \frac{\varTheta }{\partial _x \varTheta }\right) ^{3}_{d}$ is given by the elements

$$\begin{aligned} \mathcal {B}(\theta ^{(a,0)} \theta ^{(b,0)} \theta ^{(c,0)} ) = u^{(a,0)} \theta ^{(b,0)} \theta ^{(c,0)} - u^{(b,0)}\theta ^{(a,0)}\theta ^{(c,0)} + u^{(c,0)}\theta ^{(a,0)}\theta ^{(b,0)}, \end{aligned}$$

for indices *a*, *b*, *c* chosen as in the basis above.

## Proof of the main theorem

Let us first reformulate our main statement in the $\theta$-formalism.

The Poisson bracket of Dubrovin–Novikov type of the form (2) corresponds to the element

12 $$\begin{aligned} \mathfrak {p}_1 = \frac{1}{2} \iint \theta \theta ^{(0,1)} \ \mathrm{d}x \mathrm{d}y \end{aligned}$$

in $\hat{\mathcal {F}}^2_1$. The bivector $\delta ^{(2k+1)}\left( x^1-y^1\right) \delta \left( x^2-y^2\right)$ corresponds to the element in $\hat{\mathcal {F}}^2_{2k+1}$ given by

$$\begin{aligned} \mathfrak {p}_{2k+1} = \frac{1}{2} \iint \theta \theta ^{(2k+1,0)} \ \mathrm{d}x \mathrm{d}y. \end{aligned}$$

Therefore, the normal form (4) in $\theta$-formalism corresponds to the element

13 $$\begin{aligned} \mathfrak {p}(c) = \mathfrak {p}_1 + \sum _{k\geqslant 1} c_k \mathfrak {p}_{2k+1} \end{aligned}$$

in $$\hat{\mathcal {F}}^2$$.

The proof of Theorem 1 reduces to prove the three statements listed in Remark 1.

### Proof of the statement in Remark 1.i

Our first observation is:

#### Lemma 3

The bivectors $\mathfrak {p}_{2k+1}$ with $k\geqslant 0$ are pairwise compatible, i.e.

$$\begin{aligned}{}[ \mathfrak {p}_{2n+1}, \mathfrak {p}_{2m+1} ] = 0 , \qquad n, m \geqslant 0. \end{aligned}$$

#### Proof

The Poisson bivectors $\mathfrak {p}_k$ do not depend on *u* and its derivatives; therefore, the variational derivatives w.r.t. *u* appearing in the definition of Schouten–Nijenhuis bracket are vanishing. $\square$

It clearly follows that $\mathfrak {p}(c)$ is a Poisson bivector for any choice of the constants $c=(c_1, c_2, \ldots )$.

### Proof of the statement in Remark 1.ii

Next we show that for any distinct choice of the constants $c=(c_1, c_2, \ldots )$ the corresponding bivector *P* belongs to a different equivalence class under Miura transformations.

#### Proposition 1

Let $\mathfrak {p}(c)$, resp. $\mathfrak {p}(\tilde{c})$, be the Poisson bivector of the normal form (13) corresponding to a choice $c=(c_1, c_2, \ldots )$, resp. $\tilde{c}=(\tilde{c}_1, \tilde{c}_2, \ldots )$, of constants. If the two sequences *c* and $\tilde{c}$ are not identically equal, then there is no Miura transformation of the second kind which maps $\mathfrak {p}(c)$ to $\mathfrak {p}(\tilde{c})$.

#### Proof

Assume there is a Miura transformation of the second kind mapping $\mathfrak {p}(c)$ to $\mathfrak {p}(\tilde{c})$, i.e.

$$\begin{aligned} e^{\mathrm {ad}_X} \mathfrak {p}(c) =\mathfrak {p}(\tilde{c}), \end{aligned}$$

for $X \in \hat{\mathcal {F}}^1_{\geqslant 1}$. This identity can be rewritten as

$$\begin{aligned} \left( \frac{e^{\mathrm {ad}_X} - 1}{\mathrm {ad}_X} \right) \mathrm {ad}_X \mathfrak {p}(c) = \mathfrak {p}(\tilde{c}) - \mathfrak {p}(c). \end{aligned}$$

The operator inside the brackets has the form

$$\begin{aligned} \left( \frac{e^{\mathrm {ad}_X} - 1}{\mathrm {ad}_X} \right) = 1 + \frac{1}{2} \mathrm {ad}_X + \cdots \end{aligned}$$

and therefore we can invert it. We obtain

14 $$\begin{aligned} \mathrm {ad}_X \mathfrak {p}(c) = \left( \frac{e^{\mathrm {ad}_X} - 1}{\mathrm {ad}_X} \right) ^{-1} \left( \mathfrak {p}(\tilde{c}) - \mathfrak {p}(c) \right) . \end{aligned}$$

By assumption the two sequences, *c* and $\tilde{c}$ are not identically equal, and hence there exists a smallest index *k* for which $c_k \not = \tilde{c}_k$. It follows that

$$\begin{aligned} \mathfrak {p}(\tilde{c}) - \mathfrak {p}(c) = (\tilde{c}_{k} - c_k ) \mathfrak {p}_{2k+1} + \cdots , \end{aligned}$$

where the dots denote terms of standard degree greater than $2k+1$. We conclude that $\mathrm {ad}_X \mathfrak {p}(c)$ has to vanish in standard degree less or equal to 2*k*, i.e.

15 $$\begin{aligned} ( \mathrm {ad}_X \mathfrak {p}(c) )_{\leqslant 2k} =0. \end{aligned}$$

So, the leading order term in the standard degree in (14) is

16 $$\begin{aligned} (\mathrm {ad}_X \mathfrak {p}(c))_{2k+1} = (\tilde{c}_k - c_k) \mathfrak {p}_{2k+1}. \end{aligned}$$

The key point of the proof is to prove that the lefthand side is a $\mathrm {ad}_{\mathfrak {p}_1}$ coboundary, which leads to a contradiction since we know that $\mathfrak {p}_{2k+1}$ is a non-trivial class in $H^2_{2k+1}(\hat{\mathcal {F}}, \mathfrak {p}_1)$.

Notice that the lefthand side in (16) can be written

17 $$\begin{aligned} \mathrm {ad}_{\mathfrak {p}_1} X_{2k} + \sum _{s=1}^{k-1} c_s \mathrm {ad}_{\mathfrak {p}_{2s+1}} X_{2(k-s)} = (\tilde{c}_k -c_k) \mathfrak {p}_{2k+1} \end{aligned}$$

and hence it is sufficient to prove that the sum in the lefthand side is in the image of $\mathrm {ad}_{\mathfrak {p}_1}$.

Equation (15) gives a sequence of constraints on *X*. Let us consider in particular the constraints with odd degree

$$\begin{aligned} (\mathrm {ad}_X \mathfrak {p}(c))_{2s+1}=0 ,\quad s=1, \ldots , k-1, \end{aligned}$$

which can be written

18 $$\begin{aligned} \mathrm {ad}_{\mathfrak {p}_1} X_{2s} +\sum _{l=1}^{s-1} c_{l} \mathrm {ad}_{\mathfrak {p}_{2l+1}} X_{2(s-l)} =0. \end{aligned}$$

This equation for $s=1$ simply says that $X_2$ is a cocycle w.r.t. $\mathrm {ad}_{\mathfrak {p}_1}$,

$$\begin{aligned} \mathrm {ad}_{\mathfrak {p}_1} X_2 = 0. \end{aligned}$$

By the vanishing of the Poisson cohomology $H^1_2(\hat{\mathcal {F}}, \mathfrak {p}_1$), $X_2$ is necessarily a coboundary, i.e.

$$\begin{aligned} X_2 = \mathrm {ad}_{\mathfrak {p}_1} f_1 \end{aligned}$$

for some $$f_1 \in \hat{\mathcal {F}}^0_1$$.

More generally, we have that for each $s=1, \ldots , k-1$

19 $$\begin{aligned} X_{2s} = \mathrm {ad}_{\mathfrak {p}_1} f_{2s-1}+\sum _{l=1}^{s-1} c_{l} \mathrm {ad}_{\mathfrak {p}_{2l+1}} f_{2(s-l)-1} \end{aligned}$$

for some $f_{2l-1} \in \hat{\mathcal {F}}^0_{2l-1}$, $l=1,\ldots ,2s-1$. We can prove this by induction. Let us therefore assume that (19) holds for $s=1, \ldots , t-1$ for $t \leqslant k-1$, and show that it holds for $s=t$ too. Substituting the inductive assumption in (18) for $s=t$, we get that

$$\begin{aligned} \mathrm {ad}_{\mathfrak {p}_1} \left( X_{2t} - \sum _{l=1}^{t-1} c_l \mathrm {ad}_{\mathfrak {p}_{2l+1}} f_{2(t-l)-1} \right) = 0. \end{aligned}$$

The expression inside the brackets is therefore a cocycle, which has to be a coboundary due to the triviality of $H^1_{2t}(\hat{\mathcal {F}}, \mathfrak {p}_1)$, i.e.

$$\begin{aligned} X_{2t} - \sum _{l=1}^{t-1} c_l \mathrm {ad}_{\mathfrak {p}_{2l+1}} f_{2(t-l)-1} = \mathrm {ad}_{\mathfrak {p}_1} f_{2t-1}, \end{aligned}$$

for some $f_{2t-1} \in \hat{\mathcal {F}}^0_{2t-1}$. This gives (19) for $s=t$.

Substituting (19) in (17), we get that

20 $$\begin{aligned} (\tilde{c}_k -c_k) \mathfrak {p}_{2k+1} = \mathrm {ad}_{\mathfrak {p}_1} \left( X_{2k} - \sum _{s=1}^{k-1} c_s \mathrm {ad}_{\mathfrak {p}_{2s+1}} f_{2(k-s)-1} \right) , \end{aligned}$$

up to a term that can be written as

$$\begin{aligned} \sum _{n\geqslant 2}^{k-1} \left[ \sum _{\begin{array}{c} s,l \geqslant 1\\ s+l = n \end{array}} c_s c_l \mathrm {ad}_{\mathfrak {p}_{2s+1}} \mathrm {ad}_{\mathfrak {p}_{2l+1}} \right] f_{2(k-n)-1} \end{aligned}$$

and therefore clearly vanishes. Equation (20) leads to sought contradiction.

The Lemma is proved. $\square$

### Proof of the statement in Remark 1.iii

Finally, we prove that any Poisson bivector with leading order $\mathfrak {p}_1$ given by (12) can always be brought to the form (13) by a Miura transformation of the second kind.

#### Proposition 2

Let $P \in \hat{\mathcal {F}}^2_{\geqslant 1}$ be a Poisson bivector with degree one term equal to $\mathfrak {p}_1$. Then there is a Miura transformation that maps *P* to a $\mathfrak {p}(c)$ for a choice of constants $c=(c_1, c_2, \ldots )$.

#### Proof

The Poisson bivector $P\in \hat{\mathcal {F}}^2_{\geqslant 1}$ has to satisfy $[P,P]=0$. We want to show by induction that, taking into account this equation, it is possible, by repeated application of Miura transformations, to put all terms in normal form and to remove all terms that come from the Bockstein homomorphism.

Let us denote by $\mathfrak {p}_{(s)}(c_1,\ldots ,c_{\lfloor s/2-1 \rfloor })$ a bivector of the form

$$\begin{aligned}&\mathfrak {p}_{(2k)}(c_1, \ldots ,c_{k-1}) = \mathfrak {p}_1 + \sum _{l=1}^{k-1} c_l \mathfrak {p}_{2l+1} + \sum _{l=k+1}^{2k-1} Q_l + P_{2k} + \cdots , \\&\mathfrak {p}_{(2k+1)}(c_1, \ldots ,c_{k-1}) = \mathfrak {p}_1 + \sum _{l=1}^{k-1} c_l \mathfrak {p}_{2l+1} + \sum _{l=k+1}^{2k} Q_l + P_{2k+1} + \cdots , \end{aligned}$$

for *s*, respectively, even or odd, where $Q_l \in \mathcal {B}\left( \frac{\varTheta }{\partial _x \varTheta } \right) ^3_l$ , $P_l \in \hat{\mathcal {F}}^2_l$, the dots denote higher-order terms, and

21 $$\begin{aligned}{}[\mathfrak {p}_{(s)}, \mathfrak {p}_{(s)} ]=0. \end{aligned}$$

The inductive hypothesis is valid for $s=2$; indeed, $\mathfrak {p}_{(2)}$ is exactly of the required form.

Let us now show that by a Miura transformation a Poisson bivector of the form $\mathfrak {p}_{(s)}$ can be made of the form $\mathfrak {p}_{(s+1)}$.

When $s=2k$ is even, in degree $2k+1$, Eq. (21) gives

$$\begin{aligned}{}[ \mathfrak {p}_1 , P_{2k} ] + \sum _{\begin{array}{c} 2l+m=2k \\ 1\leqslant l \leqslant k-1\\ k+1 \leqslant m \leqslant 2k-1 \end{array}} [c_l \mathfrak {p}_{2l+1}, Q_{m} ] =0. \end{aligned}$$

The first observation is that both terms above need to be separately zero. This follows from the fact that the first term has nonzero degree in the number of derivatives w.r.t. *y*, while the second term has degree zero.

By Corollary 1, the cohomology $H^2_{2k}(\hat{\mathcal {F}})$ is given only by elements coming from the Bockstein homomorphism, and therefore exists $Q_{2k} \in \mathcal {B}\left( \frac{\varTheta }{\partial _x \varTheta } \right) ^3_{2k}$ such that $P_{2k} +\mathrm {ad}_{\mathfrak {p}_1} X_{2k-1} = Q_{2k}$ for some $X_{2k-1} \in \hat{\mathcal {F}}^1_{2k-1}$.

Acting with the Miura transformation $e^{\mathrm {ad}_{X_{2k-1}}}$ on $\mathfrak {p}_{(2k)}$, we get a new Poisson bivector, where the terms of degree less or equal to $2k-1$ are unchanged, the term $P_{2k}$ has been replaced with the term $Q_{2k}$, and the terms of higher order are in general different. We have therefore that $\mathfrak {p}_{(2k+1)} = e^{\mathrm {ad}_{X_{2k-1}}} \mathfrak {p}_{(2k)}$ is of the form above, as required.

When $s=2k+1$ is odd, in degree $2k+2$ from (21) we get

22 $$\begin{aligned}{}[ \mathfrak {p}_1, P_{2k+1} ] + \sum _{\begin{array}{c} 2l+m=2k+1 \\ 1 \leqslant l \leqslant k-1 \\ k+1 \leqslant m \leqslant 2k \end{array} } [c_l \mathfrak {p}_{2l+1} , Q_m] +\frac{1}{2} [Q_{k+1},Q_{k+1}] =0 . \end{aligned}$$

As in the previous case, the first term has to vanish; hence, $P_{2k+1}$ is an $\mathrm {ad}_{\mathfrak {p}_1}$-cocycle. The cohomology $H^2_{2k+1}(\hat{\mathcal {F}})$ decomposes in two parts; therefore, there is a constant $c_k$ and an element $Q_{2k+1}$ in $\mathcal {B}\left( \frac{\varTheta }{\partial _x \varTheta } \right) ^3_{2k+1}$ such that $P_{2k+1} + \mathrm {ad}_{\mathfrak {p}_1} X_{2k} = c_k \mathfrak {p}_{2k+1} + Q_{2k+1}$ for some $X_{2k} \in \hat{\mathcal {F}}^1_{2k}$.

The second and third terms in (22) have also to be both zero. This follows from the fact that they have different degree in the number of $u^{(s,t)}$. As we have seen in Sect. 3, the elements $Q_k$ are linear in the variables $u^{(s,t)}$, while the elements $\mathfrak {p}_{k}$ do not contain them.

From the vanishing of the last term, $[Q_{k+1}, Q_{k+1}]= 0$, we finally derive that $Q_{k+1}$ is zero. This is guaranteed by Lemma 4. The proof of this Lemma, being quite technical, is given in Sect. 4.4.

Taking into account this vanishing, the action of the Miura transformation $e^{\mathrm {ad}_{X_{2k}}}$ on $\mathfrak {p}_{(2k+1)}$ gives exactly the term $\mathfrak {p}_{(2k+2)}$.

By induction, we see that we can continue this procedure indefinitely; therefore, we conclude that we cannot have any non-trivial deformation coming from $\left( \frac{\varTheta }{\partial _x \varTheta } \right) ^3$ via the Bockstein homomorphism, and that the Miura transformation $\cdots e^{\mathrm {ad}_{X_2}} e^{\mathrm {ad}_{X_1}}$ given by the composition of the Mira transformations defined above, sends the original Poisson bivector $P = \mathfrak {p}_1 + \cdots$ to a Poisson bivector of the form $\mathfrak {p}(c)$ for a choice of constants $c_1, c_2, \ldots$.

The Proposition is proved. $\square$

### Some technical lemmas

In this section, we prove the following statement, which is essential in the proof of Proposition 2:

#### Lemma 4

Let $\chi \in \left( \frac{\varTheta }{\partial _x \varTheta } \right) ^3_d$ and $\mathcal {B}(\chi )$ its image through the map (9) in $\hat{\mathcal {F}}^2_d$. If $[\mathcal {B}(\chi ),\mathcal {B}(\chi ) ]=0$, then $\chi =0$.

#### Proof

We have

23 $$\begin{aligned}{}[ \mathcal {B}(\chi ) ,\mathcal {B}(\chi ) ]&= 2 \iint \frac{\delta \mathcal {B}(\chi )}{\delta \theta } \frac{\delta \mathcal {B}(\chi )}{\delta u} = 2 \iint \frac{\delta \mathcal {B}(\chi )}{\delta \theta } \frac{\delta \chi }{\delta \theta } \nonumber \\&= - 2 \iint \mathcal {B}\left( \frac{\delta \chi }{\delta \theta } \right) \frac{\delta \chi }{\delta \theta } = - \iint \mathcal {B}\left( \frac{\delta \chi }{\delta \theta } \right) ^2, \end{aligned}$$

where the second and third equalities follow from the simple identities

$$\begin{aligned} \frac{\delta \mathcal {B}(\chi )}{\delta u} = \frac{\delta \chi }{\delta \theta } , \qquad \left[ \mathcal {B}, \frac{\delta }{\delta \theta }\right] _+ = 0. \end{aligned}$$

Since we proved that the map

$$\begin{aligned} \mathcal {B}: \left( \frac{\varTheta }{\partial _x \varTheta }\right) ^{p+1}_d \rightarrow H^p_d(\hat{\mathcal {F}}) \end{aligned}$$

is injective, the vanishing of (23) implies that $\left( \frac{\delta \chi }{\delta \theta } \right) ^2=0$ in $\left( \frac{\varTheta }{\partial _x \varTheta }\right) ^{4}$. From this fact, it follows that $\chi =0$, as we prove in the remaining part of this section.^1^

Let $\mathrm {sq}:\varTheta ^2_k\rightarrow \varTheta ^4_{2k}$ be the map that sends an element $\alpha \in \varTheta ^2_k$ to $\alpha ^2\in \varTheta ^4_{2k}$. In the rest of this section, we will use the notation $\theta ^d = \theta ^{(d,0)}$.

#### Lemma 5

The intersection of $\mathrm {sq}(\varTheta ^2_k)$ and $\partial _x\varTheta ^4_{2k-1}$ is equal to zero. In other words, if $\alpha \in \varTheta ^2_k$ and $\alpha ^2$ is $\partial _x$-exact, then $\alpha ^2=0$ and, therefore, $\alpha$ is proportional to a monomial $\theta ^i\theta ^{k-i}$ for some $i=1,\ldots ,\lfloor \frac{k-1}{2} \rfloor$.

#### Proof

A basis in $\varTheta _{2k-1}^4$ is given by standard monomials $\theta ^{i_1} \theta ^{i_2} \theta ^{i_3} \theta ^{i_4}$ with total degree $i_1 + i_2+ i_3+i_4 = 2k-1$. By *standard* monomial, we indicate a monomial where the indices are ordered as $i_1> i_2> i_3 > i_4 \geqslant 0$ to avoid duplicates.

We can write $\varTheta _{2k-1}^4 = \mathcal {V}_1 \oplus \mathcal {V}_2$, where a basis for $\mathcal {V}_1$ is given by standard monomials with the restriction $i_1+i_4 \leqslant k-1$, and a basis for $\mathcal {V}_2$ is given by standard monomials with $i_1 +i_4 \geqslant k$.

It is convenient to define also the subspace $\mathcal {W}$ of $\varTheta _{2k}^4$ which is spanned by all monomials that appear in the $\partial _x \mathcal {V}_1$; more explicitly $\mathcal {W}$ is generated by the monomials

$$\begin{aligned} \theta ^{i_1+1} \theta ^{i_2} \theta ^{i_3} \theta ^{i_4}, \quad \theta ^{i_1} \theta ^{i_2+1} \theta ^{i_3} \theta ^{i_4}, \quad \theta ^{i_1} \theta ^{i_2} \theta ^{i_3+1} \theta ^{i_4},\quad \theta ^{i_1} \theta ^{i_2} \theta ^{i_3} \theta ^{i_4+1}, \end{aligned}$$

with $$i_1> i_2> i_3 > i_4 \geqslant 0$$, $$i_1+i_4 \leqslant k-1$$, and $$i_1 + i_2+ i_3+i_4 = 2k-1$$.

We denote by $\varTheta _k^2 \cdot \varTheta _k^2$ the subspace of $\varTheta _{2k}^4$ spanned by standard monomials $\theta ^{i_1} \theta ^{i_2} \theta ^{i_3} \theta ^{i_4}$ with $i_1> i_2> i_3 > i_4 \geqslant 0$ and $i_1 + i_2+ i_3+i_4 = 2k$ with $i_1+i_4 = k$ and $i_2 + i_3 = k$. It is indeed the subspace given by the product of two arbitrary elements of $\varTheta _k^2$.

Clearly, both $\partial _x \mathcal {V}_1$ and $\varTheta _k^2 \cdot \varTheta _k^2$ are subspaces of $\mathcal {W}$.

Let us now prove that $\partial _x \mathcal {V}_2$ has zero intersection with $\mathcal {W}$. Let $v = \sum _\gamma v_\gamma \, \gamma$ be an element in $\mathcal {V}_2$, where $\gamma$ is in the standard basis of $\mathcal {V}_2$ described above. Let $\partial _x v = \sum _\gamma v_\gamma \, \partial _x \gamma \in \mathcal {W}$. We have already seen that the elements $\partial _x \gamma$ are linearly independent. If $\gamma = \theta ^{i_1} \theta ^{i_2} \theta ^{i_3} \theta ^{i_4}$ then $\partial _x \gamma$ is equal to $\theta ^{i_1+1} \theta ^{i_2} \theta ^{i_3} \theta ^{i_4}$ plus lexicographically lower terms. The lexicographically leading order term is therefore of a standard monomial $\theta ^{j_1} \theta ^{j_2} \theta ^{j_3} \theta ^{j_4}$ with $j_1+j_4 \geqslant k+1$. But all basis elements in $\mathcal {W}$ are standard monomials with $j_1+j_4 \leqslant k$. It follows that, if $\gamma$ is the lexicographically highest term in *v*, we must have $v_\gamma =0$. By induction *v* vanishes.

The two facts $\partial _x \mathcal {V}_1 \subseteq \mathcal {W}$ and $\partial _x \mathcal {V}_2 \cap \mathcal {W}= (0)$ imply at once that the preimage $\partial _x^{-1}( \mathcal {W})$ in $\varTheta _{2k-1}^4$ is contained in $\mathcal {V}_1$, and the same holds for $\varTheta _k^2 \cdot \varTheta _k^2$ since it is a subspace of $\mathcal {W}$, i.e. we have

$$\begin{aligned} \partial _x^{-1} ( \varTheta _k^2 \cdot \varTheta _k^2 ) \subseteq \mathcal {V}_1. \end{aligned}$$

Since $\mathrm {sq}(\varTheta _k^2) \subseteq \varTheta _k^2 \cdot \varTheta _k^2$, our original problem reduces to finding the intersection of $\mathrm {sq}(\varTheta _k^2)$ and $\partial _x \mathcal {V}_1$.

Let $\alpha = \sum _{i = \lceil \frac{k+1}{2} \rceil }^{k} \alpha _i\, \theta ^i \theta ^{k-i}$ be an element of $\varTheta _k^2$ whose square is in $\partial _x \mathcal {V}_1$. We want to show that at most one of the coefficients $\alpha _i$ is nonzero. We therefore assume that at least two such coefficients are nonzero and show that it leads to a contradiction. Let *s* be the higher index for which $\alpha _s \not = 0$ and $t <s$ the second higher index for which $\alpha _t \not =0$.

Denote by $\mathcal {W}^{(j)}$ the subspace of $\varTheta _k^2 \cdot \varTheta _k^2$ spanned by monomials of the form

$$\begin{aligned} \theta ^{i} \theta ^{j} \theta ^{k-j} \theta ^{k-i} \text { for } i= k, \ldots , j+1 , \end{aligned}$$

and denote by $\widetilde{\mathcal {W}}$ the space spanned by the basis monomials in $\mathcal {W}$ which are not in $\varTheta _k^2 \cdot \varTheta _k^2$. Notice that

$$\begin{aligned} \varTheta _k^2 \cdot \varTheta _k^2 = \bigoplus _{j=\lceil \frac{k+1}{2} \rceil }^{k-1} \mathcal {W}^{(j)} , \end{aligned}$$

and consequently

$$\begin{aligned} \mathcal {W}= \widetilde{\mathcal {W}} \oplus \bigoplus _{j=\lceil \frac{k+1}{2} \rceil }^{k-1} \mathcal {W}^{(j)}. \end{aligned}$$

Observe that a monomial $\theta ^{i} \theta ^{j} \theta ^{k-j} \theta ^{k-i}$ in $\mathcal {W}^{(j)}$ can appear in the $\partial _x$-image of four different monomials in $\varTheta ^4_{2k-1}$ but only two of them are elements of $\mathcal {V}_1$, i.e.

$$\begin{aligned} \theta ^{i-1} \theta ^{j} \theta ^{k-j} \theta ^{k-i} ,\quad \theta ^{i} \theta ^{j} \theta ^{k-j} \theta ^{k-i-1}, \end{aligned}$$

so we only need to consider these two.

Notice that a monomial in $\mathcal {V}_1$ of such form, i.e. $\theta ^{l} \theta ^{j} \theta ^{k-j} \theta ^{k-l-1}$ is mapped by $\partial _x$ to the sum of four monomials, two of which are in $\mathcal {W}^{(j)}$, i.e.

$$\begin{aligned} \theta ^{l+1} \theta ^{j} \theta ^{k-j} \theta ^{k-l-1}, \quad \theta ^{l} \theta ^{j} \theta ^{k-j} \theta ^{k-l}, \end{aligned}$$

and two are in $\widetilde{\mathcal {W}}$.

Since $\alpha ^2 \in \varTheta _k^2 \cdot \varTheta _k^2$, it can be decomposed in its components $(\alpha ^2)_j \in \mathcal {W}^{(j)}$, and we have in particular that

$$\begin{aligned} (\alpha ^2)_t = 2 \alpha _s \alpha _t \, \theta ^s \theta ^t \theta ^{k-t} \theta ^{k-s} , \end{aligned}$$

since we have assumed that $\alpha _i=0$ for $i>s$ and $t<i<s$.

All these observations imply that there must be an element $\beta$ of $\mathcal {V}_1$ of the form

$$\begin{aligned} \beta = \sum _{i=k-1}^{t+1} \beta _i \theta ^{i} \theta ^{t} \theta ^{k-t} \theta ^{k-i-1} \end{aligned}$$

such that its image through $\partial _x$ gives $(\alpha ^2)_t$ plus some element in $\widetilde{\mathcal {W}}$.

The lexicographically higher term in $\beta$, i.e. for $i=k-1$, is sent by $\partial _x$ to a term proportional to $\theta ^{k} \theta ^{t} \theta ^{k-t} \theta ^{0}$, which does not appear in $(\alpha ^2)_t$, therefore $\beta _{k-1}=0$. Proceeding like this, we set to zero all the constants $\beta _{k-1}, \ldots , \beta _{s}$. Similarly, we can proceed from the lower part of the chain and set to zero all the remaining constants $\beta _{t+1}, \ldots , \beta _{s-1}$. But then $\beta =0$, therefore $\alpha _s \alpha _t =0$ and we are led to a contradiction.

We have proved that at most one of the constants $\alpha _i$ can be nonzero. In such case, $\alpha ^2 =0$. The Lemma is proved. $\square$

#### Lemma 6

Consider an arbitrary element $\chi \in \varTheta ^3_d$. If $\frac{\delta \chi }{\delta \theta } = c\cdot \theta ^i\theta ^{d-i}$ for some $i=0,1,\ldots ,\lfloor \frac{d-1}{2}\rfloor$, then $c=0$.

#### Proof

Consider the basis of $\left( \frac{\varTheta }{\partial _x \varTheta } \right) ^3_d$ given in Lemma 2, and the basis

$$\begin{aligned} \theta ^d\theta ^0,\theta ^{d-1}\theta ^1,\theta ^{d-2}\theta ^2,\ldots \end{aligned}$$

of $\varTheta ^2_d$. For this choice of bases, the map $\frac{\delta }{\delta \theta }$ has a two-step triangular structure. In order to explain that, let us consider the two cases of odd and even *d* separately.

Consider first the $d=2k+1$ case. One can check^2^ that the variational derivative $\frac{\delta }{\delta \theta }$ of a basis element $\theta ^{k-l+1} \theta ^{k-l} \theta ^{2l}$, with $3l < k$, is equal to

$$\begin{aligned} 2 (-1)^{k-l+1} \theta ^{d-2l} \theta ^{2l} + (d-2l) (-1)^{k-l+1} \theta ^{d-2l-1} \theta ^{2l+1} \end{aligned}$$

plus terms which are of lower lexicographic order. Notice that the coefficients of the two monomials above are non-vanishing.

Observe that $\frac{\delta }{\delta \theta } \theta ^{k+1}\theta ^k\theta ^0$ contains the monomials $\theta ^d\theta ^0$ and $\theta ^{d-1}\theta ^1$, while the variational derivatives of all other basis elements with $l\geqslant 1$ cannot contain $\theta ^d\theta ^0$ and $\theta ^{d-1}\theta ^1$. Thus, if $\frac{\delta \chi }{\delta \theta } = c\cdot \theta ^i\theta ^{d-i}$ for some *i*, then the coefficient of $\theta ^{k+1}\theta ^k\theta ^0$ in $\chi$ has to be equal to zero.

We can continue this process by induction. Assume that we have already proved that the first *l* elements of the basis cannot appear in $\chi$. Then the variational derivative of the basis element $\theta ^{k-l+1} \theta ^{k-l} \theta ^{2l}$ is the only one that contains $\theta ^{d-2l} \theta ^{2l}$ and $\theta ^{d-2l-1} \theta ^{2l+1}$. It follows from the same reason as above, that such basis element cannot appear in $\chi$.

In the case $d=2k$, we can apply the same reasoning. In this case, the variational derivative $\frac{\delta }{\delta \theta }$ of a basis element $\theta ^{k-l} \theta ^{k-l-1} \theta ^{2l+1}$, with $3l < k-2$, is equal to

$$\begin{aligned} 2 (-1)^{k-l} \theta ^{d-2l-1} \theta ^{2l+1} + (d-2l-1) (-1)^{k-l} \theta ^{d-2l-2} \theta ^{2l+2} \end{aligned}$$

plus terms of lower lexicographic order. Notice that $\theta ^d\theta ^0$ never enters the image of any basis element in $\varTheta ^3_{d}/\partial _x\varTheta ^{3}_{d-1}$. Since the coefficients of the two monomials above are non-vanishing, we can apply the same argument as in the case of odd *d*, mutatis mutandis. $\square$

Now let us consider an arbitrary element $\chi \in \varTheta ^3_d$, such that $(\frac{\delta \chi }{\delta \theta } )^2$ belongs to the image of $\partial _x$. From Lemma 5, it follows that $\frac{\delta \chi }{\delta \theta } =c\cdot \theta ^i\theta ^{d-i}$ for some $i=0,1,\ldots ,\lfloor d/2\rfloor$. Then Lemma 6 implies that $\frac{\delta \chi }{\delta \theta } =0$; hence, $\chi$ belongs to the image of $\partial _x$.

We have proved that $\chi =0$ as element of $\left( \frac{\varTheta }{\partial _x \varTheta } \right) ^3_d$. Lemma 4 is proved. $\square$

## The numerical invariants of the Poisson bracket

In principle all the numerical invariants of a Poisson bracket of the form (1), namely the sequence $(c_1,c_2,\ldots )$, can be extracted iteratively solving order by order for the Miura transformation which eliminates the coboundary terms. Providing a general formula for the invariants of a Poisson bivector is hard, since the elimination of each coboundary term affects in principle all the higher-order ones and it is necessary to give an explicit form for the Miura transformation. However, the lowest invariants can be computed as follows.

### Proposition 3

Consider a Poisson bracket of the form

$$\begin{aligned}&\left\{ u\left( x^1,x^2\right) , u\left( y^1,y^2\right) \right\} \\&\quad =\left\{ u\left( x^1,x^2\right) , u\left( y^1,y^2\right) \right\} ^0 \\&\qquad \quad +\sum _{k>0} \epsilon ^k \sum _{\begin{array}{c} k_1,k_2 \geqslant 0 \\ k_1 +k_2 \leqslant k+1 \end{array}} A_{k; k_1, k_2}(u(x)) \delta ^{(k_1)}\left( x^1-y^1\right) \delta ^{(k_2)}\left( x^2-y^2\right) , \end{aligned}$$

as in (1). Here $A_{k; k_1,k_2} \in \mathcal {A}$ and $\deg A_{k; k_1,k_2} = k - k_1 - k_2 +1$. Then the first numerical invariants of the bracket, giving the normal form of Theorem 1, are

24 $$\begin{aligned} c_1&=A_{2;3,0}, \end{aligned}$$

25 $$\begin{aligned} c_2&=A_{4;5,0}(u)-A_{2;3,0}A_{2;2,1}(u). \end{aligned}$$

Notice that $A_{2;3,0}$ is implied to be a constant.

### Proof

We recall that, given a Poisson bracket *P* of form (1), it can be expanded according to its differential order. For notational compactness, we will denote

$$\begin{aligned} P_{k+1}:=\sum _{\begin{array}{c} k_1,k_2 \geqslant 0 \\ k_1 +k_2 \leqslant k+1 \end{array}} A_{k; k_1, k_2}(u(x)) \delta ^{(k_1)}\left( x^1-y^1\right) \delta ^{(k_2)}\left( x^2-y^2\right) \end{aligned}$$

for $k>0$, so that $\deg P_k=k$.

In this proof, we replace $(x^1,x^2)$ with (*x*, *y*) as we did in the previous sections; moreover, with a slight abuse of notation we identify the Dirac’s delta derivatives with the corresponding elements of $\hat{\mathcal {F}}$ previously used

$$\begin{aligned} \mathfrak {p}_1&:=\delta \left( x^1-y^1\right) \delta ^{(1)}\left( x^2-y^2\right)&\mathfrak {p}_k&:=\delta ^{(k)}\left( x^1-y^1\right) \delta \left( x^2-y^2\right) . \end{aligned}$$

Using this notation, the Schouten identity $[P,P]=0$ reads

26 $$\begin{aligned} 2[\mathfrak {p}_1,P_k]+\sum _{l=2}^{k-1}[P_l,P_{k-l+1}]=0 \end{aligned}$$

for $k\geqslant 2$. The first equation is $[\mathfrak {p}_1,P_2]=0$; we solved it in [2], finding for $P_2$

$$\begin{aligned} A_{1;2,0}&=0&A_{1;1,1}&=0&A_{1;0,2}&=0\\ A_{1;1,0}&=-f(u)\partial _{y}u&A_{1;0,1}&=f(u)\partial _{x}u&A_{1;0,0}&=0 \end{aligned}$$

for any function *f*(*u*). Since $H^2_2(\hat{\mathcal {F}})=0$, we have $P_2=[X_1,\mathfrak {p}_1]$ and the Miura transformation that eliminates $P_2$ from *P* is $e^{-\mathrm {ad}_{\epsilon X_1}}$. The evolutionary vector field $X_1$ has characteristic

$$\begin{aligned} X_1(u)= F(u)\partial _{x}u \end{aligned}$$

where $F(u)=\int ^u f(s) ds$. We also observe that $\mathrm {ad}_{X_1}^m\mathfrak {p}_1=0$ for $m>1$.

We apply the Miura transformation generated by $-\epsilon X_1$ to *P* and get

$$\begin{aligned} \tilde{P}=e^{-\mathrm {ad}_{\epsilon X_1}}P&=\mathfrak {p}_1+\epsilon ^2 P_3+\epsilon ^3\left( P_4-[X_1,P_3]\right) \\&\quad +\epsilon ^4\left( P_5-[X_1,P_4]+\frac{1}{2}[X_1,[X_1,P_3]]\right) +\cdots \end{aligned}$$

The first equation of the system (26) for $\tilde{P}$, and the results used in the proof of Lemma 2 give us $P_3=c_1\mathfrak {p}_3+[X_2,\mathfrak {p}_1]$.

$[X_2,\mathfrak {p}_1]$ is a bivector whose degree in the number of derivatives w.r.t. $x^2$ is at least 1; notice that $x^1$ corresponds to *x* and $x^2$ corresponds to *y*, in the notation of Sects. 3 and 4. Hence, we can write

$$\begin{aligned} P_3&=A_{2;3,0}(u)\mathfrak {p}_3+A_{2;2,1}(u)\delta ^{(2)}\left( x^1-y^1\right) \delta ^{(1)}\left( x^2-y^2\right) \\&\quad +A_{2;1,2}(u)\delta ^{(1)}\left( x^1-y^1\right) \delta ^{(2)}\left( x^2-y^2\right) +A_{2;0,3}(u)\delta \left( x^1-y^1\right) \delta ^{(3)}\left( x^2-y^2\right) \\&\quad +\cdots \\&=c_1\mathfrak {p}_3+[X_2,\mathfrak {p}_1] \end{aligned}$$

This equation immediately gives $A_{2;3,0}(u) = A_{2;3,0} = c_1$ as in (24). Moreover, we can solve it for $X_2$; the characteristic of the evolutionary vector field which is a differential polynomial with top degree w.r.t. the *x* derivatives is $1/2\,A_{2;2,1}(u)\partial _{x}^2u+\tilde{A}(u)\left( \partial _{x}u\right) ^2$. Here we are interested only in first summand because it is the one that gives the highest number of *x*-derivatives in $[X_2,\mathfrak {p}_r]$, for any *r*.

We apply to $\tilde{P}$ the Miura transformation $e^{-\mathrm {ad}_{\epsilon ^2 X_2}}$ to eliminate the coboundary term of $P_3$ and are left with

27 $$\begin{aligned} e^{-\mathrm {ad}_{\epsilon ^2X_2}}\tilde{P}= & {} \mathfrak {p}_1+\epsilon ^2c_1\mathfrak {p}_3+\epsilon ^3\left( P_4-c_1[X_1,\mathfrak {p}_3]-[X_1,[X_2,\mathfrak {p}_1]]\right) \nonumber \\&+\,\epsilon ^4\left( P_5-[X_1,P_4]+\frac{1}{2}c_1[X_1,[X_1,\mathfrak {p}_3]]+\frac{1}{2}[X_1,[X_1,[X_2,\mathfrak {p}_1]]]\right. \nonumber \\&\left. -\,c_1[X_2,\mathfrak {p}_3]-\frac{1}{2}[X_2,[X_2,\mathfrak {p}_1]]\right) +\cdots \end{aligned}$$

We now use the fact that $H^2_4(\hat{\mathcal {F}})=0$ to get

$$\begin{aligned} P_4=c_1[X_1,\mathfrak {p}_3]+[X_1,[X_2,\mathfrak {p}_1]]+[X_3,\mathfrak {p}_1] \end{aligned}$$

for some homogeneous vector field $X_3$ of degree 3. This allows us to replace $P_4$ in (27) and to apply the Miura transform $e^{-\mathrm {ad}_{\epsilon ^3 X_3}}$ to it to get rid of the term $\epsilon ^3$ in the expansion. The terms of order $<3$ are left unaffected by this transformation, while the coefficient of $\epsilon ^4$ becomes

$$\begin{aligned}&P_5-[X_1,[X_3,\mathfrak {p}_1]]-\frac{1}{2}c_1[X_1,[X_1,\mathfrak {p}_3]]-\frac{1}{2}[X_1,[X_1,[X_2,\mathfrak {p}_1]]]\\&\quad -c_1[X_2,\mathfrak {p}_3]-\frac{1}{2}[X_2,[X_2,\mathfrak {p}_1]]=c_2\mathfrak {p}_5+[X_4,\mathfrak {p}_1] \end{aligned}$$

where the equality is given by our results about $H^2_5(\hat{\mathcal {F}})$ and the proof of Lemma 2. The invariant $c_2$ must be read taking the coefficient of $\mathfrak {p}_5$ in the lefthand side of the equation: this coefficient cannot be obtained by summands that are of *y*-degree bigger or equal to 1. Thus we focus on the summands

$$\begin{aligned} P_5-\frac{1}{2}[X_1,[X_1,\mathfrak {p}_3]]-c_1[X_2,\mathfrak {p}_3]=c_2\mathfrak {p}_5+\cdots . \end{aligned}$$

A direct computation shows that in $\mathrm {ad}_{X_1}^2\mathfrak {p}_3$ the term $\mathfrak {p}_5$ does not appear, while it does appear in $[X_2,\mathfrak {p}_3]$. Using the form of $X_2$ we have previously derived, we find

$$\begin{aligned} P_5=(A_{4;5,0}(u)\mathfrak {p}_5+\cdots )=\left( c_2+c_1A_{2;2,1}(u)\right) \mathfrak {p}_5+\cdots \end{aligned}$$

from which we get (25). $\square$

### Example 2

We can compute all the numerical invariants when the Poisson bracket is particularly simple. Let us consider the bracket

28 $$\begin{aligned} \{u(x),u(y)\}= & {} \delta \left( x^1-y^1\right) \delta '\left( x^2-y^2\right) +\delta '''\left( x^1-y^1\right) \delta \left( x^2-y^2\right) \nonumber \\&+\,\delta ''\left( x^1-y^1\right) \delta '\left( x^2-y^2\right) . \end{aligned}$$

Proposition 3 immediately tells us that $c_1=1$ and $c_2=-1$. Let us denote for brevity $\mathfrak {p}_{s,t}$ the bivector corresponding to $\frac{1}{2}\int \theta \theta ^{(s,t)}$. The bivector corresponding to the bracket then reads $P=\mathfrak {p}_1+\mathfrak {p}_3+\mathfrak {p}_{2,1}$, and $\mathfrak {p}_{2,1}=\mathrm {ad}_{X_2}\mathfrak {p}_1$. It is very easy to derive $X_2=\frac{1}{2}u_{2x}\theta$. We have $\mathrm {ad}_{X_2}\mathfrak {p}_{s,t}=\mathfrak {p}_{s+2,t}$. The Miura transformation $e^{-\mathrm {ad}_{X_2}}$ applied to *P* gives

$$\begin{aligned} P_{(1)}&=\mathfrak {p}_1+\sum _{n=0}^\infty \frac{(-1)^n}{n!}\mathrm {ad}_{X_2}^n\mathfrak {p}_3+\sum _{n=1}^\infty (-1)^n\left( \frac{1}{n!}-\frac{1}{(n+1)!}\right) \mathrm {ad}_{X_2}^{n+1}\mathfrak {p}_1 \\&=\mathfrak {p}_1+\sum _{n=0}^\infty \frac{(-1)^n}{n!}\mathfrak {p}_{3+2n}+\sum _{n=1}^\infty (-1)^n\left( \frac{1}{n!}-\frac{1}{(n+1)!}\right) \mathfrak {p}_{2n+2,1} \end{aligned}$$

Notice that the term $n=0$ in the first sum gives the only contribution of order 3, giving $c_1=1$. The further $\mathfrak {p}_1$-coboundary term should be read in the $n=1$ term of the second sum, namely for $-\frac{1}{2}\mathfrak {p}_{4,1}=\mathrm {ad}_{X_4}\mathfrak {p}_1$. The next Miura transformation leads to

$$\begin{aligned} P_{(2)}&=\mathfrak {p}_1+\sum _{m=0}^\infty \sum _{n=0}^\infty \frac{(-1)^{n+2m}}{2^m m!n!}\mathfrak {p}_{2n+4m+3}+\sum _{m=1}^\infty \frac{(-1)^{2m}}{2^mm!}\mathfrak {p}_{4m,1}\\&\quad +\sum _{m=0}^\infty \sum _{n=2}^\infty \left( \frac{1}{n!}-\frac{1}{(n-1)!}\right) \frac{(-1)^{n+2m}}{2^mm!n!}\mathfrak {p}_{2n+4m,1}. \end{aligned}$$

The procedure goes on—always requiring us to find the vector field cancelling the lowest order term of the form $\mathfrak {p}_{s,1}$. At each step, we will need vector fields $X_{2s+2}$ such that

$$\begin{aligned} \mathrm {ad}_{X_{2s}}\mathfrak {p}_1=\frac{(-1)^{s+1}}{s}\mathfrak {p}_{2s,1} \end{aligned}$$

and we obtain

$$\begin{aligned} P_{(\infty )}=\left( \prod _{s=1\ldots \infty }^{\curvearrowleft } e^{-\mathrm {ad}_{X_{2s}}}\right) \,P. \end{aligned}$$

The Miura transformation cancels all the terms of the form $\mathfrak {p}_{s,1}$ and we are left with the following expression for the Poisson bivector brought to the normal form:

$$\begin{aligned} P_{(\infty )}=\mathfrak {p}_1+\sum _{m_1,m_2,\ldots =0}^{\infty }\frac{(-1)^{m_1+2m_2+3m_3+\cdots }}{m_1!m_2!m_3!\cdots 2^{m_2}3^{m_3}\cdots }\mathfrak {p}_{3+2m_1+4m_2+6m_3+\cdots } \end{aligned}$$

We recall that $\frac{1}{2}\int \theta \partial _x^k\theta =\mathfrak {p}_k$. Hence, the infinite sum can be seen as a series expansion for $\frac{1}{2}\int \theta \partial _x^3/(1+\partial _x^2)\,\theta$ as follows:

$$\begin{aligned} \frac{1}{2}\int \theta \partial _x^3\left( \sum _{m_1=0}^\infty \frac{(-1)^{m_1}}{m_1!}\partial _x^{2m_1}\right) \left( \sum _{m_2=0}^\infty \frac{(-1)^{2m_2}}{2^{m_2}m_2!}\partial _x^{4m_2}\right) \left( \sum _{m_3=0}^\infty \frac{(-1)^{3m_3}}{3^{m_2}m_3!}\partial _x^{6m_3}\right) \cdots \theta \\= \frac{1}{2}\int \theta \left( \partial _x^3\, e^{-\partial _x^2+\frac{\partial _x^4}{2}-\frac{\partial _x^6}{3}+\cdots }\right) \theta =\frac{1}{2}\int \theta \,\partial _x^3\, e^{-\log (1+\partial _x^2)}\theta =\frac{1}{2}\int \theta \,\frac{\partial _x^3}{1+\partial _x^2}\theta . \end{aligned}$$

We stress the fact that all these identities should always been understood in terms of formal power expansion. On the other hand, a more obvious expansion for the same expression is

$$\begin{aligned} \frac{1}{2}\int \theta \,\frac{\partial _x^3}{1+\partial _x^2}\theta =\frac{1}{2}\int \theta \partial _x^3\sum _{k=0}^\infty (-1)^k\partial _x^{2k}\theta , \end{aligned}$$

that translates into

$$\begin{aligned} P_{(\infty )}=\mathfrak {p}_1-\sum _{k=1}^\infty (-1)^k\mathfrak {p}_{2k+1} , \end{aligned}$$

and gives us all the numerical invariants of (28).

### Example 3

(Hamiltonian structure of Helmoltz’s equation) Helmoltz’s equation describes the time evolution of the vorticity in an ideal incompressible fluid [17]. In the two-dimensional case, it is an evolutionary equation for the scalar vorticity of the fluid $\omega (x,y)$ with velocity field $\mathbf {v}(x,y)=(u(x,y),v(x,y))$, given by

$$\begin{aligned} \omega _t=-u\, \omega _x - v \,\omega _y. \end{aligned}$$

The vorticity of the fluid is the scalar quantity $\omega =v_x-u_y$. Such an equation is not integrable, but it is Hamiltonian [23] with respect to the functional

$$\begin{aligned} H=\frac{1}{2}\int \left( u^2+v^2\right) \end{aligned}$$

and the Poisson bracket

29 $$\begin{aligned} \{\omega (x,y),\omega (w,z)\}=\omega _x\delta (x-w)\delta ^{(1)}(y-z)-\omega _y\delta ^{(1)}(x-w)\delta (y-z). \end{aligned}$$

It should be noticed that also in this case, as for Example 1, the Hamiltonian functional is not local in the field $\omega$. Indeed, the incompressibility of the fluid in two dimensions allows us to introduce the *stream function* $\psi (x,y)$ such that $u=\psi _y$ and $v=-\psi _x$, for which $\varDelta \psi =-\omega$ and $\delta H/\delta \omega =-\psi$.

The bracket (29) is not of the form (1); however, it is compatible with the bracket $\{\omega (x,y),\omega (w,z)\}^{0}=\delta (x-w)\delta ^{(1)}(y-z)$, which allows us to consider the first-order deformation given by the Poisson bivector

30 $$\begin{aligned} P=\mathfrak {p}_1+\frac{1}{2}\int \left( u_x\theta \theta ^{(0,1)}-u_y\theta \theta ^{(1,0)}\right) . \end{aligned}$$

The deformation is a coboundary in the Poisson cohomology of $\mathfrak {p}_1$, which follows from $H^2_2(\mathfrak {p}_1)=0$. In particular, it is obtained by $[\mathfrak {p}_1,X_1]$ with $X_1=-uu_x\theta$. Moreover, a simple computation shows that $[X_1,[X_1,\mathfrak {p}_1]]=0$. This means that with a Miura transformation $\exp ^{-X_1} P=\mathfrak {p}_1$, and the normal form of the bracket (30) has numerical invariants $c_k\equiv 0$.

## Footnotes

1 Notice that this fact, in the case of standard differential polynomials in commuting variables, follows from a simple observation: the derivative in *x* of a differential polynomial cannot be a square, since it has to be linear in the highest derivative. In the case of anticommuting variables, however, a quite involved proof is necessary.

2 Note that the computation is slightly different in the case $3l=k-1$.
